# Psychosocial Impacts of Idiopathic Clubfoot on Parents and Children: A Scoping Review Protocol

**DOI:** 10.3390/healthcare12181871

**Published:** 2024-09-18

**Authors:** Nurhanis Syazni Roslan, Syurahbil Abdul Halim, Ismail Munajat, Sarina Sulong

**Affiliations:** 1 Department of Medical Education, School of Medical Sciences, Universiti Sains Malaysia, Kubang Kerian 16150, Kelantan, Malaysia; 2 Department of Orthopaedics, School of Medical Sciences, Universiti Sains Malaysia, Kubang Kerian 16150, Kelantan, Malaysia; ismailmu@usm.my; 3 Human Genome Centre, School of Medical Sciences, Universiti Sains Malaysia, Kubang Kerian 16150, Kelantan, Malaysia; ssarina@usm.my

**Keywords:** compliance, idiopathic clubfoot, psychosocial, quality of life, talipes

## Abstract

Background/Objectives: Idiopathic clubfoot is a complex pediatric foot deformity. The Ponseti technique is widely regarded as the standard for correcting deformities, and treatment compliance is essential for preventing relapse. Examining psychosocial effects on parents and/or children during clubfoot treatment provides valuable insights for improving compliance. This scoping review will map the existing literature on the psychological and social effects experienced by parents and/or children with idiopathic clubfoot. It also aims to examine the assessment tools used to measure these impacts and identify factors influencing treatment compliance. Methods: This review will adhere to the Joanna Briggs Institute (JBI) guidelines for scoping reviews. The search will include databases such as Scopus, Web of Science, EBSCOhost, MEDLINE, and PsycINFO and focus on studies published in the last 10 years. This review will include quantitative, qualitative, and mixed-method studies that investigate the psychological and social effects experienced by parents or affected children of any age with idiopathic clubfoot. Reporting will follow the Preferred Reporting Items for Systematic Reviews and Meta-Analyses extension for Scoping Reviews (PRISMA-ScR) extension guidelines. The screening and data extraction process will involve two independent reviewers. The analysis will be descriptive and qualitative. Results: The findings will be presented in tables and a narrative summary. Conclusion: This review may guide health practitioners in developing evidence-based interventions to improve treatment adherence.

## 1. Introduction

Clubfoot, also known as talipes equinovarus, is the most common complex neonatal foot deformity, with four distinct components: forefoot adductus, midfoot cavus, hindfoot equinus, and varus [1]. Based on the main underlying pathology, clubfoot can be divided into two main categories: idiopathic or congenital and non-idiopathic or syndromic. Of these, the most common is the idiopathic type, where the cause remains unclear. Genetic links to the pathogenesis of idiopathic clubfoot have been the subject of recent research; however, the evidence remains inconclusive [2]. It is thought that environmental factors also play an essential role in the development of idiopathic clubfoot [3]. Non-idiopathic clubfoot is associated with disorders such as arthrogryposis, myelodysplasia, or other associated congenital deformities [4]. The incidence of idiopathic clubfoot varies between 1 and 2 cases for every 1000 live births, influenced by factors such as ethnicity and geographical location [5,6]. A total of 50% of idiopathic clubfoot is bilateral [2].

Failure to receive early treatment can have detrimental effects on a child’s developmental milestones, particularly in achieving normal walking abilities. Structural alterations in the foot will progressively become permanent, accompanied by the development of soft tissue contractures [4,6]. In the case of a rigid foot, the likelihood of successful correction through casting diminishes, increasing the probability of the child requiring surgical correction [2].

The preferred treatment method for idiopathic clubfoot is the Ponseti technique, involving gradual correction through weekly casting [5]. Correction follows a specific order: addressing cavus, then adductus, varus, and finally equinus. Numerous studies, exploring the effectiveness of the Ponseti technique, have indicated success rates of 90–100% for children under 2 years old and around 90% for those aged between 1 and 3 years [7,8]. Though it is recommended to initiate treatment as early as possible [4], the specific age when treatment begins does not significantly influence the clinical outcomes [9]. A previous study that looked at the optimal age for initiating casting proposed that initiation between 28 days and 3 months of age resulted in a lower recurrence rate [8].

In cases where residual equinus remains after the weekly casting regimen is completed, approximately 90% of children may need to undergo Achilles tenotomy, a surgical intervention, to fully correct the equinus. Following this, the child wears a long leg cast for 3 more weeks, with the foot in a dorsiflexed and abducted position [2,10]. Subsequently, during the maintenance phase, the child is put in an abduction brace to prevent relapse [2,6]. Once the initial deformity correction has been achieved, the abduction brace is applied for 23 h a day during the first 3 months, followed by night-time usage for a duration of 3 to 4 years [11].

Adherence to the Ponseti protocol directly impacts the outcomes [5,12]. Compliance with abduction bracing leads to fewer recurrences and surgeries [12,13]. The main causes of non-compliance in the majority of cases were attributed to parental factors [14,15]. In their study of 108 patients, Jawadi et al. [15] discovered that factors contributing to non-compliance included parents dealing with unilateral clubfoot, those responsible for caring for three or more children, and difficulty seeing their child cry while in the brace. A study in Nigeria revealed 15.5% and 12.0% prevalences of emotional strain and parental burden in caregivers of children with clubfoot, respectively [16]. The frequency of parental stress was higher when taking care of younger children with clubfoot [16]. A critical factor in maintaining brace adherence is the parents’ understanding of its importance and correct application, as they are the ones responsible for its use at home. Building a strong family partnership and fostering commitment, alongside in-depth educational sessions about brace application, are pivotal elements in ensuring compliance [14,15,17].

In the current literature, several systematic reviews have been conducted on various aspects of clubfoot, including the outcomes of the Ponseti method, recurrence rates of deformities, and orthotic interventions [4,18,19,20]. However, systematic reviews specifically addressing health-related quality of life remain limited, and existing reviews do not focus on idiopathic clubfoot [21,22]. After conducting a preliminary search in MEDLINE, PubMed, Scopus, Cochrane Database of Systematic Reviews, and JBI Evidence Synthesis, we found that there are currently no ongoing or existing scoping reviews focusing on this topic.

This scoping review aims to answer the following review questions: (1) What are the psychosocial impacts of idiopathic clubfoot on the well-being of both parents and children with idiopathic clubfoot? (2) What assessment tools are used to measure the psychosocial impact of idiopathic clubfoot on both parents and children diagnosed with idiopathic clubfoot? (3) What are factors contributing to treatment compliance in parents and children with idiopathic clubfoot?

## 2. Materials and Methods

The proposed scoping review will be conducted following the Joanna Briggs Institute (JBI) methodology for scoping reviews [23]. The Preferred Reporting Items for Systematic Reviews and Meta-Analyses extension for Scoping Reviews (PRISMA-ScR) checklist will be used as a guideline for the report [24]. This review will follow five steps for scoping reviews: (1) identifying review questions, (2) searching relevant studies, (3) selecting eligible studies, (4) data extraction and charting, and (5) analyzing and reporting the results [25]. This study protocol has been registered in the Open Science Framework (https://osf.io/ut3ey, accessed on 12 September 2024).

### 2.1. Search Strategy

The search strategy will aim to locate published studies only. Unpublished studies or gray literature will not be considered owing to time and resource constraints. A preliminary limited search of Scopus, Web of Science, EBSCOhost, PubMed, and PsycINFO with the search strategy using (clubf**t OR equinovarus) AND (psychosocial OR factor OR social OR “quality of life” OR “life quality” OR tool OR scale OR instrument OR questionnaire) was undertaken to identify articles on the topic. The text words contained in the titles and abstracts of the relevant articles and the index terms used to describe the articles were used to develop a full search strategy for Scopus, Web of Science, EBSCOhost, MEDLINE (Ovid), and PsycINFO (Appendix A: Table A1). The search strategy, including all identified keywords and index terms, will be adapted for each database and/or information source. Eligible studies will be restricted to those published in English since 2013 (over the last 10 years). The reference lists of all included sources of evidence will be screened for additional studies. Additional relevant articles from the reference lists of the included articles will be included if they meet the inclusion criteria (Table 1).

### 2.2. Inclusion Criteria

#### 2.2.1. Participants

This review will include studies on either the parents of children with idiopathic clubfoot or children of any age who were diagnosed with idiopathic clubfoot and underwent any treatment including non-surgical intervention such as serial casting, orthosis, or abduction brace, and surgical treatment such as Achilles tenotomy or soft tissue release. Idiopathic clubfoot is defined as foot deformities characterized by forefoot adductus, midfoot cavus, and hindfoot equinus and varus, and with unknown etiology [1]. This review will include studies with newly diagnosed, neglected, or relapse cases of idiopathic clubfoot. Studies focusing on pediatric patients with non-idiopathic or syndromic clubfoot will be excluded. As the review will map psychosocial themes related to treatment, studies examining the long-term impact on adults who had idiopathic clubfoot as children will not be included. Parents can be defined as both parents or either one of the parents or caretakers. There will be no restrictions on the age, ethnicity, or gender of the parents.

#### 2.2.2. Concept

This review will include studies exploring the psychosocial impacts of the diagnosis and treatment of idiopathic clubfoot on parents and children affected by the deformity. In this study, psychological impacts will be defined as the direct effects of idiopathic clubfoot on the psychological/mental, emotional, and social functioning of the parents/caretakers and the affected children. The impacts will be captured from the perspectives of both parents/caretakers and children and potentially include the effect on siblings. Parental viewpoints include mental well-being, socio-interpersonal dynamics, family interactions, and the overall quality of life. A child’s perspective includes well-being, developmental milestones, familial and social interactions, and quality of life. This review also will analyze the assessment tools used to examine psychosocial impacts. Additionally, it will include studies exploring the factors influencing treatment compliance and their potential interplay with psychosocial determinants. Studies on idiopathic clubfoot with no description of the psychosocial components, tools, or contributing factors will be excluded.

#### 2.2.3. Context

For eligible studies, there will be no restrictions on geographical location, socioeconomic status, cultural or subcultural background, or demographic characteristics based on race or gender. Furthermore, this review will consider all techniques and modalities for non-surgical intervention in idiopathic clubfoot, as well as surgical intervention.

### 2.3. Type of Sources

This scoping review will consider quantitative, qualitative, or mixed-methods study design. It will also include both experimental and quasi-experimental study designs, such as randomized controlled trials, non-randomized controlled trials, before-and-after studies, and interrupted time-series studies. In addition, analytical observational studies, including prospective and retrospective cohort, case-control, and analytical cross-sectional studies, will be considered for inclusion. Descriptive observational study designs, including case series, individual case reports, and descriptive cross-sectional studies, will be considered for inclusion. Qualitative studies that concentrate on qualitative data will be evaluated for inclusion. This includes, but is not limited to, designs such as phenomenology, grounded theory, ethnography, qualitative descriptions, action research, and feminist research. Conference abstracts will be considered depending on the availability of the full text or slide presentations. Reviews, expert opinions, and letters to editors will be excluded from this scoping review. This scoping review will only consider studies published in English.

### 2.4. Selection of Sources

Following the search, all identified citations will be collated and uploaded into Microsoft Excel version 16.83 and duplicates will be removed. Following a pilot test, titles and abstracts will then be screened by two independent reviewers (N.S.R. and S.A.H.) to assess the inclusion criteria for the review. Potentially relevant sources will be retrieved in full text, and their citation details will be imported into a Microsoft Excel spreadsheet. The full text of the selected citations will be assessed in detail against the inclusion criteria by two independent reviewers (N.S.R. and I.M.). Reasons for excluding sources of evidence in the full text that do not meet the inclusion criteria will be recorded and reported in the scoping review. Any disagreements between the reviewers at each stage of the selection process will be resolved through discussion or by a third reviewer (S.S.). The results of the search and study inclusion process will be reported in full in the final scoping review and presented in a PRISMA-ScR flow diagram [24].

### 2.5. Data Extraction

Data from the included studies will be incorporated into a data extraction sheet developed by the reviewers using Microsoft Excel version 16.83. The extracted data will include descriptions of any psychological and social factors experienced by one or both parents and, when reported, by children with idiopathic clubfoot. The review will also extract the description of assessment tools used to measure the psychosocial impact of treatment of idiopathic clubfoot on the parents and, when reported, on the children themselves, and any factors related to parental compliance with the Ponseti serial casting protocol. A draft of the data extraction form is provided (Appendix B: Table A2). The draft will be modified and revised as necessary during the process of extracting data from each of the included evidence sources. Modifications will be reported in the scoping review publication.

### 2.6. Data Analysis and Presentation

The data will be analyzed using descriptive and qualitative methods. Study characteristics will be converted into standardized categorical data using frequency counts. Qualitative data will include content or thematic analysis [26]. A narrative summary will be used to describe the results in relation to the review questions.

## 3. Results

**Figure 1:**
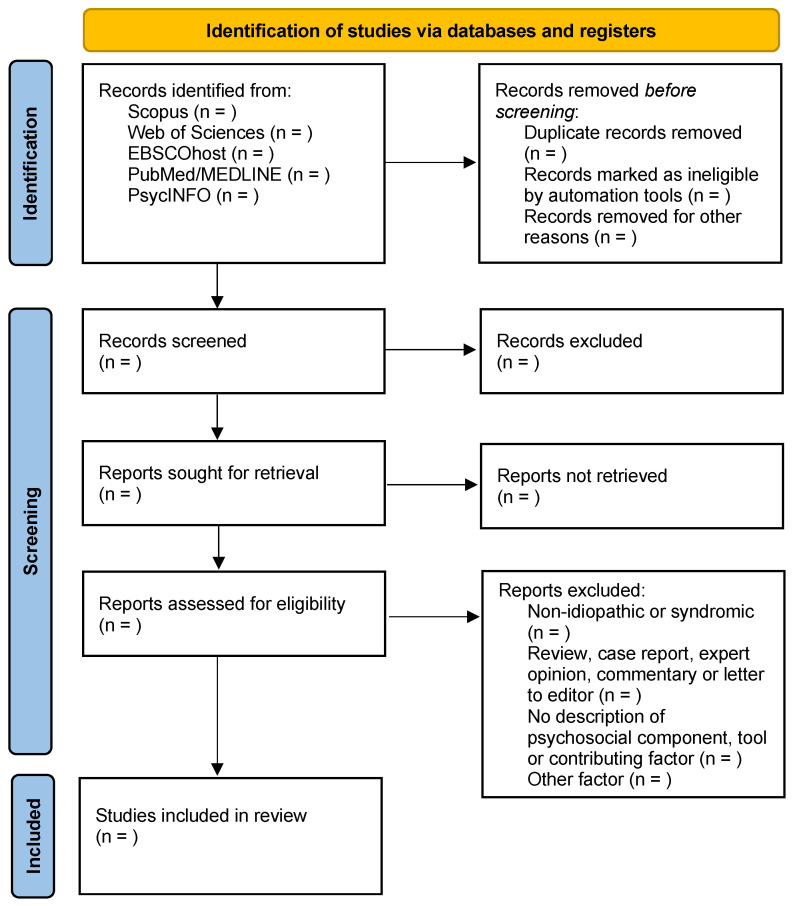
PRISMA flow diagram to summarize search results, study selection, and inclusion process.

The search results will be synthesized and reported in a PRISMA flow diagram, as illustrated in Figure 1 [27]. The data will be presented in a tabular format. All included studies will be presented in tables, including the authors’ names, year of publication, country, study design, number of participants, psychosocial impact, assessment tools, and treatment compliance factors. A narrative summary will be used to describe each table.

## 4. Discussion

Children with idiopathic clubfoot often face more than just physical challenges. A small subset of these children may also experience neurodevelopmental difficulties that can impact their overall quality of life [28,29]. Despite the recognized impact of idiopathic clubfoot on children’s well-being and the experiences of their parents/caregivers, there is a lack of comprehensive synthesis of the literature exploring these psychosocial aspects. Thus, this scoping review aims to systematically map the most recent literature concerning the psychological and social impacts of idiopathic clubfoot diagnosis and treatment in both parents and, when reported, children with the condition. Additionally, it seeks to outline the assessment tools employed to measure these psychological and social impacts and investigate how these psychosocial factors influence treatment adherence. 

This review findings may guide health practitioners in developing holistic and evidence-based intervention strategies to improve treatment compliance, particularly with serial casting protocols and abduction braces. These strategies can facilitate successful deformity correction while minimizing the likelihood of relapse since treatment compliance has been identified as a significant predictive determinant for clubfoot recurrence [30,31].

Caring for a child with congenital deformities or anomalies that require extensive treatment can significantly increase the parental psychological burden [16,32,33]. This emotional burden was found to be particularly elevated when the child was between 3 and 5 years old, a critical developmental period marked by increased physical and social demands [16]. Studies have shown that mothers often experience more pronounced emotional distress compared to fathers when providing care for a child with congenital limb deformities [16,33]. In the case of idiopathic clubfoot, parental psychological concerns may arise from negative emotional responses toward the diagnosis, the daily challenges of caregiving, and uncertainty about the child’s future [34].

The findings from this scoping review may have important implications for future clinical practice. The identified psychosocial factors could provide a foundation for developing assessment tools that healthcare providers can routinely use when evaluating patients with idiopathic clubfoot. Timely referrals to appropriate support services may help improve compliance with the Ponseti casting treatment protocol. Additionally, identifying unique psychosocial domains associated with idiopathic clubfoot treatment could allow further research, development, and evaluation of psychosocial interventions aimed at improving compliance and the success of casting treatment and follow-up.

This scoping review has several key strengths. It will comprehensively examine the fundamental psychosocial impacts experienced by children with congenital deformities, such as idiopathic clubfoot, and their families. Given that the treatment requires a substantial amount of childhood time to correct the deformity and support normal child development, this review will potentially capture the important psychosocial domains that warrant specific focus from a wide range of health providers. Additionally, the use of a comprehensive search strategy across multiple general and subject-specific databases, including Scopus, Web of Science, EBSCOhost, MEDLINE, and PsycINFO, will help maximize the identification of all relevant studies. Another key strength is that the review will include both quantitative and qualitative studies, aiming to capture the diverse experiences of affected children and their parents/caregivers.

While this scoping review aims to provide a comprehensive overview of the psychosocial impacts of idiopathic clubfoot, it is essential to acknowledge certain potential limitations. One limitation is the restriction to studies published only in English, which may exclude potentially relevant research conducted in other languages. Additionally, the screening of studies for inclusion and the extraction of data may involve some degree of subjectivity, potentially leading to variation in interpretation. To mitigate this limitation, the review will employ multiple reviewers to conduct the study screening and data extraction processes, with any disagreements resolved through discussion or with the assistance of a third reviewer.

## 5. Conclusions

This scoping review aims to systematically map the current literature on the psychological and social experiences of parents of children with idiopathic clubfoot, as well as the experiences of the affected children themselves. Identifying the key psychosocial factors impacting these individuals is essential for understanding treatment compliance and informing the development of future evidence-based interventions, research, clinical practice, or health policy related to the management of idiopathic clubfoot.

## Figures and Tables

**Table 1:** Inclusion and exclusion criteria used during screening of articles in the scoping review.

	Inclusion	Exclusion
Participants	Either parents of children with idiopathic clubfoot or children who were diagnosed with idiopathic clubfoot and underwent any treatment (any age, gender, or ethnicity) Newly diagnosed, neglected, or relapse cases	Pediatric patients with non-idiopathic or syndromic clubfoot Adults who had idiopathic clubfoot as children
Concept	Psychosocial impacts of the diagnosis and treatment of idiopathic clubfoot ∘ Parental viewpoints include mental well-being, socio-interpersonal dynamics, family interactions, and the overall quality of life. ∘ Child’s view includes well-being, developmental milestones, familial and social interactions, and quality of life. Assessment tools used to examine psychosocial impacts Factors influencing treatment compliance and potential interplay with psychosocial determinants	No description of the psychosocial components, tools, or contributing factors
Context	Any geographical location, socioeconomic status, cultural or subcultural background, or demographic characteristics based on race or gender Any techniques of non-surgical and surgical intervention	
Types of sources	Quantitative, qualitative, or mixed-methods study design including the following: ∘ Experimental or quasi-experimental studies ∘ Analytical observational studies ∘ Descriptive observational study designs Conference abstracts—with full text or slide presentations English language	Reviews, expert opinions, and letters to editors

## Data Availability

The data from this study are available upon request.

## Appendix A

The following is a preliminary search strategy for two databases (PubMed and Scopus) conducted on 20 August 2024.

**Table A1:** Search conducted in two of the selected databases using Advanced Search (performed on 20 August 2024).

#	Query	PubMed	Scopus
1	clubf**t [Title/Abstract] OR equinovarus [Title/Abstract]	4657	8576
2	(clubf**t [Title/Abstract] OR equinovarus [Title/Abstract]) AND ((“2013” [Date—Publication]: “3000” [Date—Publication]))	1757	3205
3	(psychosocial or factor or social or “quality of life” or “life quality” or tool or scale or instrument or questionnaire) AND (psychosocial [Title/Abstract] OR factor [Title/Abstract] OR social [Title/Abstract] OR “quality of life” [Title/Abstract] OR “life quality” [Title/Abstract] OR tool [Title/Abstract] OR scale [Title/Abstract] OR instrument [Title/Abstract] OR questionnaire [Title/Abstract])	11034247	21976556
4	((psychosocial OR factor OR social OR “quality of life” OR “life quality” OR tool OR scale OR instrument OR questionnaire) AND (psychosocial [Title/Abstract] OR factor [Title/Abstract] OR social [Title/Abstract] OR “quality of life” [Title/Abstract] OR “life quality” [Title/Abstract] OR tool [Title/Abstract] OR scale [Title/Abstract] OR instrument [Title/Abstract] OR questionnaire [Title/Abstract])) AND ((“2013” [Date—Publication]: “3000” [Date—Publication]))	5378248	12152656
5	((clubf**t [Title/Abstract] OR equinovarus [Title/Abstract]) AND ((“2013” [Date—Publication]: “3000” [Date—Publication]))) AND (((psychosocial or factor or social OR “quality of life” OR “life quality” OR tool OR scale OR instrument OR questionnaire) AND (psychosocial [Title/Abstract] OR factor [Title/Abstract] OR social [Title/Abstract] OR “quality of life” [Title/Abstract] OR “life quality” [Title/Abstract] OR tool [Title/Abstract] OR scale [Title/Abstract] OR instrument [Title/Abstract] OR questionnaire [Title/Abstract])) AND ((“2013” [Date—Publication]: “3000” [Date—Publication])))	362	1022

## Appendix B

**Table A2:** Draft data extraction instrument.

Author(s)	
Year of publication	
Country	
Study objective	
Study design	
Participants number	
Psychological impacts	
Assessment tool	
Treatment compliance factors	
